# Cerebrovascular Disease in the Setting of Posterior Reversible Encephalopathy Syndrome

**DOI:** 10.3389/fneur.2021.765333

**Published:** 2021-11-17

**Authors:** XiaoQing Cheng, JianRui Li, Ying Lan, Jia Liu, Sui Chen, GuangMing Lu

**Affiliations:** ^1^Department of Medical Imaging, Jinling Hospital, Nanjing University School of Medicine, Nanjing, China; ^2^Special Medical Service, Lushan Rehabilitation and Recuperation Center of People's Liberation Army (PLA), Jiujiang, China

**Keywords:** posterior reversible encephalopathy syndrome, reversible cerebral vasoconstriction syndrome, magnetic resonance imaging, blood brain barrier, angiography, diffusion-weighted imaging

## Abstract

Overlap between the pathogenesis of posterior reversible encephalopathy syndrome and that of cerebrovascular disease can confound their clinical and radiological presentations, posing a diagnostic challenge. This article presents a literature review and discussion of the clinical manifestations, pathological mechanisms, and imaging manifestations of subarachnoid hemorrhage and vasculitis leading to posterior reversible encephalopathy syndrome, coexistence of posterior reversible encephalopathy syndrome with reversible cerebral vasoconstriction syndrome, and hemorrhage and infarction secondary to posterior reversible encephalopathy syndrome. The findings show that posterior reversible encephalopathy syndrome shares some overlapping pathophysiological mechanisms with cerebrovascular disease. Importantly, neuroimaging plays an important role in identifying this entity in a timely manner and differentiating it from other diseases.

## Introduction

Posterior reversible encephalopathy syndrome (PRES) is a reversible acute neurological disorder characterized by varied neurological symptoms, including seizure, headache, focal neurological deficit, visual disturbance, and altered consciousness (1–3). Case series have shown that hypertension, eclampsia, renal failure, systemic lupus erythematosus, and use of some immunosuppressive agents are common causes of PRES (1–4). Brain MRI, particularly fluid-attenuated inversion recovery is the most sensitive sequence for detecting PRES, and usually reveals characteristic vasogenic edema predominantly affecting subcortical white matter of the parietal and occipital lobes (5, 6).

Among the various theories that have been proposed for the pathogenesis of PRES, hypertension and endothelial injury are often cited as the underlying mechanisms (1). Rapidly developing hypertension exceeds the upper limit of cerebral blood flow autoregulation and causes hyperperfusion, which leads to breakdown of the blood–brain–barrier and subsequent vasogenic brain edema (7, 8). Thus, multiple interactions exist between PRES and cerebrovascular diseases, such as subarachnoid hemorrhage (SAH) and vasculitis (9, 10), reversible cerebral vasoconstriction syndrome (RCVS) (11–13), and cerebral hemorrhage and infarction secondary to PRES (5, 14–16).

Overlap between PRES and cerebrovascular disease in terms of their clinical and pathological mechanisms and imaging manifestations can easily lead to misdiagnosis, differences in treatment modalities, and a poor prognosis when secondary hemorrhage and infarction are indicated. Therefore, it is important to understand and identify PRES associated with cerebrovascular disease (Table 1). In this review, we discuss PRES and multiple cerebrovascular diseases in terms of their clinical features, pathological mechanisms, and imaging manifestations.

**Table 1:** PRES and cerebrovascular disease.

	**PRES secondary to SAH and vasculitis**	**PRES coexisting with RCVS**	**Hemorrhage and infarction secondary to PRES**
Incidence	1. Incidence of PRES among patients with SAH after induced hypertension therapy is 1.7–7% 2. PRES secondary to TA has been reported in 13 patients	RCVS is present in ~8–85% of patients with PRES	1. The incidence of intracranial hemorrhage secondary to PRES was 15.2–64.5% 2. The incidence of cerebral infarction secondary to PRES is 9%
Pathogenesis	1. Ruptured aneurysm causing a sudden increase in blood pressure and BBB breakdown after induced hypertension therapy; 2. Panarteritis involving vessel wall layers	1. Abnormal cerebral autoregulation 2. BBB breakdown 3. Vascular endothelial damage	1. Vessel rupture 2. Reperfusion injury 3. Cytotoxic edema
Imaging	1. Vasospasm and PRES can be differentiated by time of onset, age, blood pressure and perfusion imaging 2. Hemodynamic changes provided by perfusion imaging can help screen patients with SAH for induced hypertension therapy 3. TA combined with intracranial artery stenosis, intracranial aneurysm, carotid artery occlusion and other vascular changes	1. The distribution of edema secondary to RCVS is different from the typical PRES lesions 2. Follow-up CTA or MRA can assess reversible vascular changes in RCVS 3. MR vessel wall imaging may be helpful in distinguishing RCVS from vasculitis 4. Perfusion imaging may be a useful approach in identifying cerebral hypoperfusion secondary to RCVS	1. Intracranial hematoma secondary to PRES occurs within the area of brain parenchyma affected by edema 2. SAH typically secondary to PRES occurs in the cerebral convexities overlying vasogenic edema and is rarely seen in basal cisterns 3. Decreased ADC values represent cytotoxic edema and can help predict infarction and irreversible tissue damage

*ADC, apparent diffusion coefficient; BBB, blood-brain barrier; RCVS, reversible cerebral vasoconstriction syndrome; PRES, posterior reversible encephalopathy syndrome; SAH, subarachnoid hemorrhage*.

## PRES Secondary to SAH and Vasculitis

### Clinical Features

SAH is most commonly caused by rupture of an intracranial aneurysm (17). After SAH, delayed cerebral ischemia (DCI) may occur when cerebral perfusion falls below the level required to meet metabolic demands, and this could lead to the development of cerebral infarction. Induced hypertension therapy raises blood pressure to above normal levels and results in an increase in cerebral blood flow; it remains the mainstay of treatment for DCI (18). However, in patients treated with induced hypertension therapy, an elevation in arterial blood pressure may exceed the threshold for autoregulatory function of the brain and may lead to spontaneous angioedema (19).

To date, PRES has been reported in 20 cases as a consequence of induced hypertension therapy, and 80% of the patients were female (19). Allen et al. (18) reported induced hypertension therapy in 68 patients with SAH, of which 5 patients (7%) were diagnosed with PRES, which most often occurred when mean arterial pressure was raised well above baseline to levels that exceed traditional autoregulatory thresholds. According to the latest data reported by Angermann et al. (9) showing that the incidence of PRES among patients with SAH after induced hypertension therapy is 1.7%, the authors noted that no safe upper limit of mean arterial pressure has been established during the treatment of vasospasm with induced hypertension therapy.

Takayasu's arteritis (TA) is a rare chronic inflammatory disease that primarily affects the aorta, aortic branches, and the pulmonary artery (10). PRES secondary to TA has been reported in 13 patients, according to a PubMed search (10, 20). In addition to TA secondary to PRES, in isolated cases, PRES has also been reported to occur secondary to rare vasculitis, such as cerebral amyloid angiopathy-related inflammation (21), and antineutrophil cytoplasmic antibody-associated vasculitis (22).

### Pathological Mechanisms

According to the literature, the pathophysiological mechanism of PRES caused by induced hypertension therapy for SAH is unclear, and it is speculated that multiple factors may be responsible. On the one hand, acute hypertension caused by catecholamine surge during aneurysm rupture stimulates the sympathetic nerves, leading to a sudden increase in blood pressure, which may induce autonomic vasoconstriction and lead to ischemia and vasogenic edema in the affected area (23). Another hypothesis is that induced hypertension therapy may increase intravascular hydrostatic pressure over the damaged blood–brain barrier and cause the rupture of vascular endothelial junctions, leading to the development of acute PRES (19, 24). Accordingly, it has been found that the percentage change in blood pressure is significantly greater in patients with PRES than in those without PRES, and that an increase in mean arterial pressure to 50 mm Hg above the patients' physiological levels or to an absolute range of >130–140 mm Hg is a sensitive predictor of PRES (18).

The main pathophysiological change in TA is panarteritis involving all vessel wall layers. In the acute phase, production of inflammatory cytokines and mediators induces continuous endothelial injury, while in the chronic phase, hyperplasia of the vessel wall and fibrosis of the arterial wall cause luminal narrowing and can lead to the development of hypertension (20). Endothelial injury and hypertension in patients with TA make this disease an ideal environment for PRES development. Furthermore, the treatment of TA begins with control of acute arteritis with immunosuppressive drugs; however, their use is also a possible factor that contributes to PRES development (9).

### Imaging Manifestations

As PRES is treated differently from vasospasm, and both may occur secondary to SAH, it is crucial to identify the two in a timely manner. Vasospasm usually develops 3–4 days after SAH and continues for 10–14 days (25). Yet, PRES is most often delayed, occurring ~1 week after induced hypertension therapy. Moreover, PRES is more likely to occur in older patients and in patients with a mean arterial pressure of 50 mmHg above habitual levels (or absolute levels of >130–140 mmHg) (18). CT perfusion imaging helps to identify delayed cerebral ischemia related to vasospasm, which can manifest as decreased cerebral blood flow and a prolonged mean transit time in the blood supply area of affected vessels (26). However, CT and MRI perfusion studies in PRES are contradictory, with two patterns: hyperperfusion and hypoperfusion. Hyperperfusion shows increased cerebral blood flow and cerebral blood volume and decreased time to peak and mean transit time, the mechanism behind which may be severe hypertension exceeding the limits of vascular autoregulation, leading to hyperperfusion (27, 28). Hypoperfusion possibly occurs due to vasoconstriction as a compensatory mechanism for hypertension, leading to decreased cerebral blood flow, near-normal cerebral blood volume, and increased time to peak and mean transit time (29, 30). The conflicting results likely reflect the complex pathophysiology of PRES. Therefore, although perfusion imaging cannot completely distinguish delayed cerebral ischemia from PRES by assessing hyperperfusion and hypoperfusion, assessing whether vasospasm leads to hemodynamic changes can help screen patients for induced hypertension therapy, avoiding blind lowering of blood pressure, which can lead to PRES development.

PRES secondary to TA presents as fluid-attenuated inversion recovery hyperintensities on MRI, while there is usually no diffusion restriction on DWI (10, 20). The onset could be in the bilateral parieto-occipital lobes, bilateral temporoparietal-occipital lobes, and the cerebellum, as reported for PRES caused by other factors (10, 20). In addition, after CTA or MRA, TA should be considered when combined intracranial artery stenosis, intracranial aneurysm, carotid artery occlusion, and other vascular changes are found.

## PRES Coexisting With RCVS

### Clinical Features

RCVS is a clinical and radiological syndrome characterized by “thundering” headaches, transient, multifocal and segmental cerebral arterial vasoconstriction lasting weeks to months, and focal neurological symptoms that may also complicate ischemic or hemorrhagic stroke (13). Although RCVS is frequently reported in the literature, most studies on RCVS are observational and thus the etiology, and the underlying mechanism remain largely unknown. The occurrence of PRES with coexisting RCVS is not uncommon in clinical practice. Studies report that RCVS is present in ~8–85% of patients with PRES who undergo MRA or catheter angiography (11–13). The diagnostic criteria for RCVS emphasize acute onset of “thundering” headache with reversible, multisegmental cerebral vasoconstriction on imaging, while excluding other diagnoses such as aneurysmal SAH or central nervous system vasculitis.

### Pathological Mechanisms

The main pathophysiological mechanisms for coexistence of PRES and RCVS include blood–brain barrier breakdown and abnormal cerebral autoregulation (3). Considering that RCVS occurs in the postpartum period and after vasoactive drug use, it is presumed that hormonal changes and drug-induced vascular endothelial damage are more directly responsible for RCVS occurrence with PRES. In addition, both vasoconstriction and hypoperfusion improve with increasing maturity of the involved brain region; thus, it has been suggested in the literature that children might be more prone to PRES and its complications (31).

RCVS has high hemorrhagic complications, some researchers attributed the high rate of hemorrhagic complications in RCVS to a reperfusion injury following the episode of severe vasoconstriction (32), and it has also been suggested that subarachnoid hemorrhage actually preceded typical segmental vasoconstriction revealed by DSA (33). However, in clinical observational studies, a causal relationship between subarachnoid hemorrhage and RCVS seems difficult to prove.

### Imaging Manifestations

**Figure 1:**
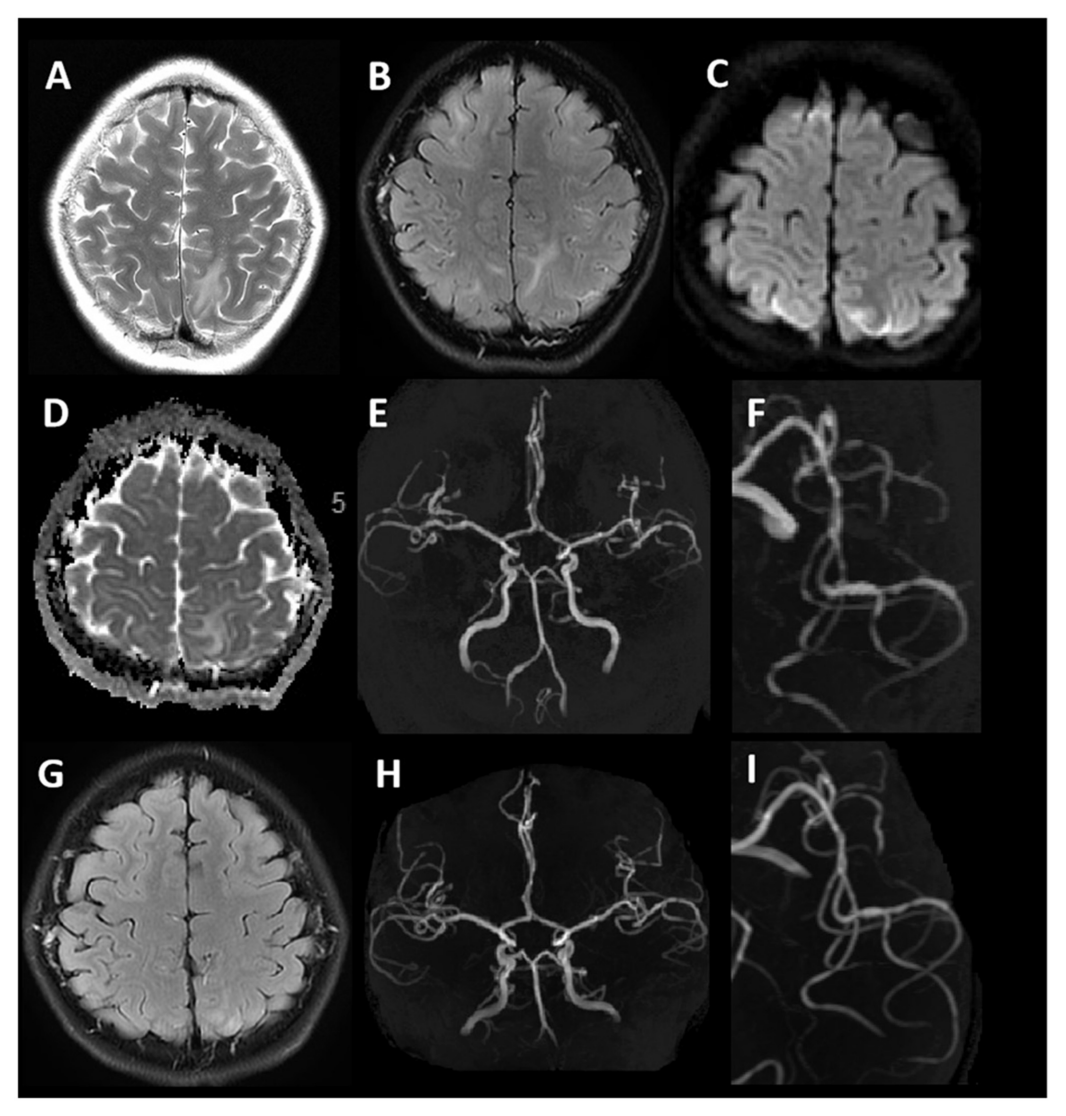
Typical MRI findings in PRES coexisting with RCVS. A 12-year-old female with lupus nephritis. T2WI and T2 -FLAIR imaging **(A,B)** show hyperintense signals involving bilateral subcortical white matter of the frontal, parietal, and temporal lobes. DWI **(C)** shows hyperintense signals in bilateral frontoparietal lobes. The corresponding ADC map **(D)** shows no diffusion restriction. MRA **(E,F)** demonstrates segmental vasoconstriction in the left middle cerebral artery branch. Follow-up MRI after 10 days (T2-FLAIR) **(G)** demonstrates complete resolution of signal abnormalities. MRA **(H,I)** demonstrates normalization of vessel irregularity.

Radiological presentations, taken together with clinical context and symptoms, may help to reach a differential diagnosis. On MRI, the typical lesion distribution in patients with PRES is characterized by bilateral symmetrical parieto-occipital lesions, whereas vasogenic edematous changes secondary to RCVS are mostly distributed in periventricular white matter and lack cortical involvement (Figure 1). Of note, it has been reported in the literature that PRES in children exhibits more of a suprafrontal sulcus pattern, while the typical parieto-occipital pattern is less frequent in children compared with adults (4). Furthermore, patients with RCVS do not usually present with seizures or severe cerebral or brainstem edema (34). From the angiographic presentation, digital subtraction angiography is the gold standard for the diagnosis of vascular lesions, while CT angiography and MRA allow for non-invasive assessment of reversible vascular changes and facilitate follow-up review (12) (Figures 1E,F,H,I). In addition, MR vessel wall imaging may be helpful in distinguishing RCVS from vasculitis, since RCVS demonstrates arterial wall thickening with absent to minimal enhancement of vessel walls compared with more pronounced enhancement in vasculitis (35). Perfusion imaging may be a useful approach in identifying cerebral hypoperfusion secondary to RCVS and to assess radiographic improvement after treatment (36).

When PRES and RCVS coexist, given the complicated disease course, the appropriate therapy is often controversial (37). Usual treatment for PRES in the setting of hypertension is to gradually lower blood pressure. However, in the setting of RCVS, an argument could be made to maintain or elevate blood pressure. Chung et al. (38) proposed the use of near-infrared spectroscopy, which is a non-invasive modality to monitor regional cerebral oxygenation and guide subsequent decision making. Therefore, the purpose of imaging is not to completely differentiate between RCVS and PRES, but to exclude other diseases and provide appropriate treatment recommendations and predict prognosis by assessing cerebral hemodynamics and cerebral oxygen saturation.

## Hemorrhage and Infarction Secondary to PRES

### Clinical Features

**Figure 2:**
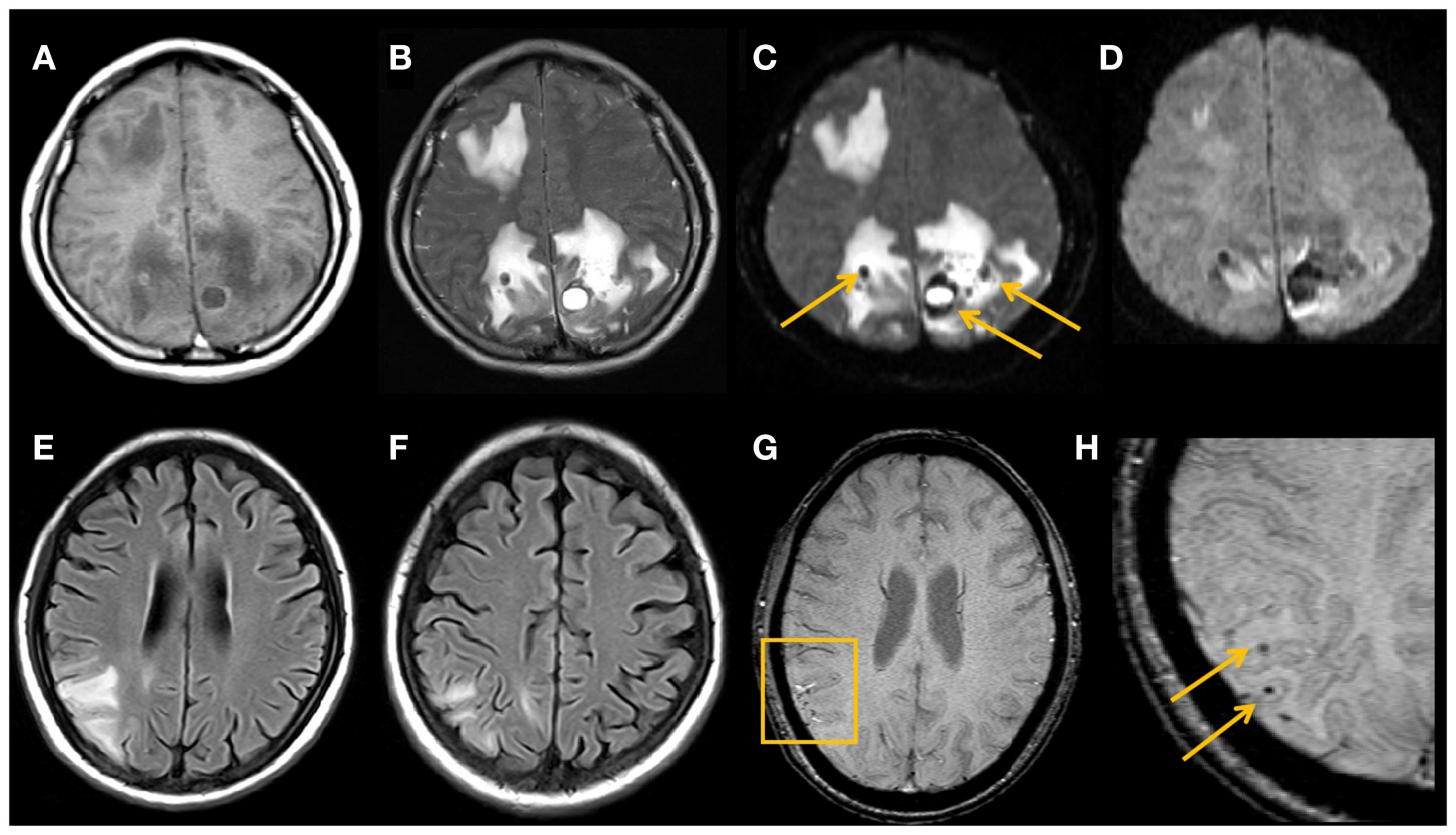
Typical MRI findings in hemorrhage secondary to PRES.Imaging studies in a 29-year-old female with lupus **(A–D)**. MRI shows vasogenic edema in the right frontal lobe and bilateral parieto-occipital lobes, multiple hematomas are seen in the area affected by the edema, with heterogeneous high signal on T1WI **(A)**, low signal on T2WI **(B)**, low signal on DWI b = 0 images (arrows) **(C)**, and magnetically sensitive artifacts of high signal around the hematoma visible on DWI b = 1,000 images **(D)**. An 18-year-old young female with nephrotic syndrome **(E–H)**. PRES-related right parieto-occipital cortical and subcortical edema on MRI FLAIR image **(E,F)**, with multiple small microhemorrhages on SWI (arrows) **(G,H)**.

Intracranial hemorrhage is a common complication of PRES, with an incidence of 15.2–64.5% (14, 15), and is associated with incomplete resolution of PRES (39). Intracranial hemorrhage manifests in three main patterns: intracranial hematoma, subarachnoid hemorrhage, and microhemorrhage, with intracranial hematoma being the most common (14). Susceptibility-weighted imaging (SWI) sequences are more sensitive than conventional T2 gradient-recalled echo imaging in detecting cerebral hemorrhage and microhemorrhage (Figures 2E–H), and 58% of patients with PRES combined with microhemorrhage were identified by SWI (15).

Vasogenic edema is a predominant feature of PRES, and the presence of restricted diffusion in some cases may represent the earliest irreversible sign: severe vasogenic edema progresses to cytotoxic edema, which further progresses to cerebral infarction (6). Covarrubias et al. (40) reported a group of 22 patients with PRES, 6 of whom developed diffusion abnormalities and 2 (9%) of whom showed progression to infarction at follow-up. Several studies have evaluated the clinical and radiological findings of patients with PRES and reported incomplete recovery and poor functional outcomes when PRES was associated with hemorrhage and infarction (3, 37, 41). Thus, early MRI features may be warning signs of a poor prognosis.

### Pathological Mechanisms

In terms of etiology, hemorrhage in PRES is associated with ongoing therapeutic anticoagulation, intrinsic coagulopathy, bone marrow transplantation, and thrombocytopenia (8). There are multiple underlying pathophysiological mechanisms that can cause hemorrhage in patients with PRES. Pial vessel rupture during severe hypertension, reperfusion injury in the setting of vasoconstriction, and endothelial injury directly caused by use of immunosuppressive agents have all been postulated as mechanisms leading to hemorrhagic PRES (7). In 2020, three patients with coronavirus disease 2019 compatible with hemorrhagic PRES were reported (42, 43). Available data suggest that the severe acute respiratory syndrome coronavirus 2 directly infects endothelial cells, causing damage to their lining, and thus increasing the permeability of the blood–brain barrier. In addition, further secondary hemorrhage can occur due to cytokine release syndrome resulting from liver dysfunction and depletion of coagulation factors (1).

On the basis of vasogenic edema caused by PRES, when further arterial vasospasm and endothelial injury lead to a decrease in local cerebral blood flow and hypoxia in brain tissue, further cytotoxic edema can develop, leading to infarction (40, 41).

### Imaging Manifestations

Intracranial hematoma secondary to PRES occurs within the area of the brain parenchyma affected by edema (Figures 2A–D). SAH secondary to PRES typically occurs in the cerebral convexities overlying vasogenic edema and is rarely seen in the basal cisterns; this is different from the area of distribution of SAH caused by aneurysm rupture and can help in the differential diagnosis of the two entities (44). In addition, PRES and cerebral venous sinus thrombosis have similar clinical presentations, causative factors, and imaging findings, such as vasogenic edema, diffusion restriction on DWI, and hemorrhage (45). The key to differentiating between them is the use of CT or MRI venography to exclude venous sinus thrombosis.

From an imaging perspective, DWI can help predict conversion to infarction and irreversible tissue damage (46). The hallmark of PRES lesions is a pattern of vasogenic edema, which is shown as an increased apparent diffusion coefficient (ADC), while decreased ADC values indicate cytotoxic edema that inevitably induces cell death and progression to true infarction. However, when vasogenic edema is combined with cytotoxic edema, it usually presents as small areas or short cortical gyriform foci of restricted diffusion within larger regions of vasogenic edema (7), unlike the territorial infarctions due to arterial occlusion.

## Conclusion

In conclusion, the occurrence of PRES is associated with various pathological mechanisms, such as abnormalities in brain autoregulation, blood–brain barrier breakdown, and vascular endothelial damage, which are also factors associated with the development of related cerebrovascular diseases. Neuroimaging plays an important role in revealing the pathophysiological mechanisms of PRES, differentiating PRES from other cerebrovascular diseases, guiding treatment, and predicting a poor prognosis.

## Author Contributions

XC: study design, data analysis, drafting the manuscript, and revising it critically. JLi and SC: acquisition of data, drafting the manuscript, and revising it critically. YL and JLiu: image processing, interpretation of the data, drafting the manuscript, and revising it critically. GL: study concept and design, review of all the images and advise on the findings. All authors have read and approved the manuscript.

## Funding

This work was supported by the major project of the National Natural Scientific Foundation of China (Grant Number: 81790653).

## Conflict of Interest

The authors declare that the research was conducted in the absence of any commercial or financial relationships that could be construed as a potential conflict of interest.

## Publisher's Note

All claims expressed in this article are solely those of the authors and do not necessarily represent those of their affiliated organizations, or those of the publisher, the editors and the reviewers. Any product that may be evaluated in this article, or claim that may be made by its manufacturer, is not guaranteed or endorsed by the publisher.
